# Arterial Hypertension and Skin Allergy Are Risk Factors for Progression from Dengue to Dengue Hemorrhagic Fever: A Case Control Study

**DOI:** 10.1371/journal.pntd.0003812

**Published:** 2015-05-21

**Authors:** Maria Glória Teixeira, Enny S. Paixão, Maria da Conceição N. Costa, Rivaldo V. Cunha, Luciano Pamplona, Juarez P. Dias, Camila A. Figueiredo, Maria Aparecida A. Figueiredo, Ronald Blanton, Vanessa Morato, Maurício L. Barreto, Laura C. Rodrigues

**Affiliations:** 1 Instituto de Saúde Coletiva, Salvador, Bahia, Brasil; 2 Universidade Federal de Mato Grosso do Sul, Faculdade de Medicina, Cidade Universitária, Campo Grande, Mato Grosso do Sul, Brasil; 3 Universidade Federal do Ceará, Instituto de Ciências de Saúde, Salvador, Bahia, Brasil; 4 Instituto de Ciências da Vida, Universidade Federal da Bahia, Salvador, Bahia, Brasil; 5 Case Western Reserve University, Cleveland, Ohio, United States of America; 6 Centro de Pesquisas Gonçalo Muniz, FIOCRUZ, Salvador, Bahia, Brazil; 7 London School of Hygiene and Tropical Medicine, London, United Kingdom; Pediatric Dengue Vaccine Initiative, UNITED STATES

## Abstract

**Background:**

Currently, knowledge does not allow early prediction of which cases of dengue fever (DF) will progress to dengue hemorrhagic fever (DHF), to allow early intervention to prevent progression or to limit severity. The objective of this study is to investigate the hypothesis that some specific comorbidities increase the likelihood of a DF case progressing to DHF.

**Methods:**

A concurrent case-control study, conducted during dengue epidemics, from 2009 to 2012. Cases were patients with dengue fever that progressed to DHF, and controls were patients of dengue fever who did not progress to DHF. Logistic regression was used to estimate the association between DHF and comorbidities.

**Results:**

There were 490 cases of DHF and 1,316 controls. Among adults, progression to DHF was associated with self-reported hypertension (OR = 1.6; 95% CI 1.1-2.1) and skin allergy (OR = 1.8; 95% CI 1.1-3.2) with DHF after adjusting for ethnicity and socio-economic variables. There was no statistically significant association between any chronic disease and progression to DHF in those younger than 15 years.

**Conclusions:**

Physicians attending patients with dengue fever should keep those with hypertension or skin allergies in health units to monitor progression for early intervention. This would reduce mortality by dengue.

## Author Summary

Dengue is a virus disease that has already reached more than 100 countries worldwide, transmitted by *Aedes* mosquitos, mainly *Aedes aegypti*. It is estimated that annually nearly 96 million symptomatic cases and about 22,000 deaths occur. This virus most often manifests itself in the form of Dengue Fever (DF), which in some cases progresses to severe forms, also called Dengue Hemorrhagic Fever (DHF). However, the current knowledge does not allow early prediction of which cases of DF will progress to DHF. But it is important to know the factors Involved in this process so that physicians may intervene early to prevent progression and avoid deaths. One of the proposed explanations is that preexisting comorbidities would increase the risk of progression from DF to DHF. This study showed that cases of DF associated with hypertension or skin allergy are more likely to progress to DHF. Monitoring and early appropriate clinical management of these cases can save lives.

## Introduction

The reemergence of dengue—which is now present in over 100 countries—is a global public health problem [1]. It is estimated that there are 390 million dengue infections every year worldwide, of which 96 million are symptomatic and about 22,000 fatal [2].

Dengue Fever (DF) is a mild disease; progression to dengue hemorrhagic fever/dengue shock syndrome (DHF / DSS) is relatively rare, but the case fatality rate of cases that do progress is high. Currently knowledge does not allow early prediction of which cases of dengue fever (DF) will progress to dengue hemorrhagic fever (DHF), to allow early intervention to prevent progression or to limit severity [3]. There is evidence a second heterologous dengue virus (DENV) infection is more likely to progress to severe dengue [4]. Halstead (1980) [4] proposed that the mechanism underlying DHF/DSS is antibody-dependent enhancement (ADE) of dengue virus infection. ADE seems to acts as an idiosyncratic Fc-receptor signalling [5] which in its turn would modulate the disease severity through suppressing the innate immune system (macrophages/monocytes), abrogating immune response to virus throughout inhibition of interferon transcription factors, STAT1, and NFκB complexes, decreasing nitric oxide production, and increasing interleukin 10 [6]. This would increase the numbers of infected cells, viral production per cell and cytokine production leading to vascular permeability, coagulopathy and ultimately the capillary leakage characteristic of DHF [5].

In some endemic areas, over 70% the population has antibodies to dengue virus, but the cumulative incidence of DHF is under 1% [7]. Clearly, other factors must contribute to the development of severe dengue. It is likely that the host genetic background and viral strains and serotype influence the risk of this progression. One of the proposed explanations was that preexisting comorbidities would increase the likelihood of progression from DF to DHF. The hypothesis was generated in an uncontrolled case series [8,9] and explored in two case control studies (one by our group here in Brazil and one in Singapore). Both were conducted retrospectively and both found that cases of DF were less likely than cases of DHF to report previous diabetes mellitus and allergies [10] and diabetes with hypertension [11]. We therefore decided to conduct an concurrent case control study, with recruitment of incident (rather than past) cases, for monitoring the clinical course, and collection of information as cases are diagnosed to reduce vulnerability to information bias and provide a rigorous confirmation of these initial findings.

## Methods

The objective of the study was to investigate whether specific morbidity due to chronic illnesses increased the risk of progression from DF to DHF/DSS. The study was conducted in Brazil, the country with the highest number of DF cases in the world [12]. The study area included 6 cities: Campo Grande/MS, Fortaleza/CE, Itabuna/BA, Jequié/BA (2009), Ilhéus/BA and Salvador/BA,

This was an unmatched concurrent case-control study, with cases and controls recruited in the Infectious Diseases Reference Hospitals in each of the 6 cities during epidemic years, from 2009 to 2012. In 2009, the DENV2 predominated (in the epidemics of Jequié, Ilheus and Itabuna). In 2010 (in Campo Grande) and 2011 (in Salvador) DENV1 predominated. In 2012, the predominant serotype was DENV4 (in Salvador and Fortaleza). From 2009 to 2011 had simultaneous circulation of three serotypes (DENV2, DENV3 and DENV1). From 2011, all four serotypes circulated simultaneously.

Recruitment: patients admitted in the hospitals with signs and symptoms of dengue were invited to participate in the study. During the first contact an interview was conducted, and cases of DF were accompanied by the research team physician until a final diagnosis, and those who progressed to DHF/DSS) were classified as cases and those who did not progress for DHF were classified as controls.

Case definition: Patients with dengue fever who progressed to DHF according to the WHO 1997 criteria [13]: fever, hemorrhagic manifestations, thrombocytopenia (<100×109/L) and evidence of plasma leakage (hematocrit change ≥20%, hypoproteinemia or clinical fluid accumulation), and one positive specific laboratory diagnosis for dengue.

Control definition: Patients, from the same hospital as cases, with signs and symptoms of DF (fever, headache or retroorbital pain, myalgia, arthralgia, prostration, exanthema and positive specific laboratory diagnosis for dengue [13] who did not progress to DHF.

Laboratory investigation: Platelia dengue NS1 Ag kit (Bio-Rad laboratories Marnes-le-Coquette France and/or DENV IgM Capture by ELISA Kit PanBio Queensland, Australia). Prior serologic status (IgG) of the cases and control was not investigated, so past history of Dengue was not established.

Data collection: Patients (and/or relatives) were interviewed when they arrived in the hospital, by trained interviewers, using a previously tested, standardized questionnaire to obtain demographic and biological data (name, address, age, sex self-reported skin color), socioeconomic indicators (years of schooling and family income). Clinical information included signs and symptoms of dengue, reported other health conditions (diabetes, hypertension, allergy, asthma) and use of medication for control of these illnesses. When the individual reported one of the conditions of interest, he/she was asked who made the diagnosis and the interviewer asked to see the prescription and/or packaging of any medication. Only subjects able to show packaging or prescriptions were considered to have the condition. As skin allergies have a wide spectrum, subjects were considered to have "allergy" if they reported allergy and provided evidence of having used anti-allergic medication, including a prescription.

Statistical analysis: Associations between each co-morbidity and DHF was investigated using the χ2 test (Fischer's exact test when appropriate). Stratified analyses were conducted to assess confounding; age (≥ 15 and <15 years) was investigated as an effect modifier. Logistic regression models were defined to estimate the independent association between DHF and hypertension, diabetes, allergy and asthma. STATA software, version 12 was used for the analyses.

Sample size: the necessary sample size to detect an odds ratio (OR) of 1.7 for the progression from DF to DHF, for a frequency of co-morbibity of 9.1% (for prevalence of asthma, the least common of the co-morbidities studied), with 95% precision, 80% power, with a ratio of 4 controls per case, was estimated to be 303 cases and 1212 controls,

Ethical approval was obtained from the Research Ethics Committee, Instituto de Saúde Coletiva, Federal University of Bahia, Salvador, Brazil (No. 013/03/CEP-ISC). Cases and controls gave written informed consent.

## Results

The study included 490 cases of DHF and 1,316 controls with DF. 64.5% of the cases were over 15 years of age. Nearly 60% of adult cases were female, 55% considered themselves mixed race, about 48% had income ≤ 1 minimum monthly salary (USD 239–306), 56.7% had at least ten years of schooling. Of the controls, 69.3% were over 15 years of age, 63.5% were female, 44% considered themselves mixed race, and 40.4% had a family income between 2–3 minimum salaries and 53% had 10 years or more of schooling. Cases and controls aged less than 15 years were similar in distribution, with approximately 52% female, 70% with a family income ≤1 minimum monthly salary; 65% of cases and 60% controls declared themselves to be mixed race (Table 1).

**Table 1:** Socioeconomic, demographic and comorbidity characteristics of patients with Hemorrhagic Dengue Fever (cases) and Dengue Fever (controls) living in six municipalities of Brazil 2009–2012.

Age group		≥15 years		≤15 years		
Characteristics	Case N = 316	Controls N = 912	p value	Case N = 174	Controls N = 404	p value
**Sex**						
Female	188 (59,49)	579 (63,49)		91 (52,3)	210 (51,98)	0,94
Male	128 (40,51)	333 (36,51)	0,206	83 (47,7)	194 (48,02)	
**Age**						
≤ 7	-	-		50 (28,74)	112 (27,72)	0,512
8–11	-	-	-	80 (45,97)	175 (43,31)	
12–15	-	-		44 (25,28)	117(28,96)	
**Skin color**						
Black	40 (12,7)	132 (14,67)		30 (17,54)	58 (14,5)	0,120
White	102 (32,38)	373 (41,4)	0,003	30 (17,54)	101 (25,25)	
Mixed	173 (54,92)	395 (43,89)		111 (64,91)	241 (60,25)	
**Income**						
≥1	144 (47,84)	286 (33,53)		117 (70,48)	258 (70,88)	0,774
1–3	103 (34,22)	345 (40,45)	0,000	38 (22,89)	76 (20,88)	
≥3	54 (17,94)	222 (26,03)		11(6,63)	30 (8,24)	
**Schooling**						
0–3	39 (13,31)	83 (10,35)		-	-	-
3–7	39 (13,31)	143 (17,83)	0,146	-	-	-
7–10	49 (16,72)	151 (18,83)		-	-	-
≥10	166 (56,66)	425 (52,99)		-	-	
**Hypertension**						
No	226 (71,52)	690 (75,82)	0,108	173 (99,4)	403 (99,7)	0,512
Yes	90 (28,48)	217 (23,85)		1 (0,6)	1 (0,25)	
**Allergy**						
No	251 (79,43)	164 (17,98)	0,309	123 (70,69)	304 (75,25)	0,250
Yes	65 (20,57)	748 (82,02)		51 (29,31)	100 (24,75)	
**Food Allergy**						
No	302 (95,57)	880 (96,60)	0,061	158 (90,80)	384 (95,05)	0,061
Yes	14 (4,43)	31 (3,40)		16 (9,2)	20 (4,95)	
**Respiratory Allergy**						
No	273 (86,39)	791 (86,83)	0,844	140 (80,46)	332 (82,38)	0,583
Yes	43 (13,61)	120 (13,17)		34 (19,54)	71 (17,62)	
**Skin Allergy**						
No	291 (92,09)	869 (95,49)	0,029	162 (93,64)	380 (94,06)	0,850
Yes	25 (7,91)	41 (4,51)		11 (6,36)	24 (5,94)	
**Diabetes**						
No	297 (94,59)	863 (94,84)	0,558	174 (100)	401 (99,26)	0,250
Yes	17 (5,41)	47(5,16)		-	3 (0,74)	
**Asthma**						
No	306 (96,84)	891 (98,8)	0,161	161 (92,53)	383 (95,51)	0,161
Yes	10 (3,16)	20 (2,20)		13 (7,47)	18 (4,49)	

^a^—Salvador; Ilhéus; Itabuna; Jequié; Fortaleza; Campo Grande.

Self-reported skin color, family income and skin allergy showed a statistically significant association with DHF (p<0.05) among subjects aged over 15 years (Table 2). Crude association with DHF were found with family income and also when adjusted for skin color, sex and age, for income between 2–3 minimum salaries (OR = 0.6, 95% CI 0.4–0.8); income for > 3 minimum salaries (OR = 0.5 95% CI 0.3–0.8). When each self-reported chronic disease was adjusted for ethnic and social variables, only hypertension (OR = 1.6; 95% CI 1.1–2.1) and skin allergy (OR = 1.8; 95% CI 1.1–3.2) were associated with DHF (Table 3). Association were statistically significant between DHF and self-reported skin allergies (only when medication was not used at the time of the illness, OR = 2.1, 95% CI 1.1–4.1), and with hypertension both when the subjects were using of antihypertensive drugs (OR = 1.4; 95% CI 1.1–2.0) and when they were not using antihypertensive drugs (OR = 1.8; 95% CI 1.1–3.2).

**Table 2:** Odds ratio crude and adjusted obtained by logistic regression for the association between Dengue Hemorrhagic Fever and socioeconomics and demographic variables of residents in six municipalities^a^ of Brazil, according to age group 2009–2012.

Age Group	≥15 years				<15 years			
^Characteristics^	Crude OR	CI 95%	Adjusted OR	CI95%	Crude OR	CI 95%	Adjusted OR	CI95%
**Skin Color**								
White	1.0		1,0		1.0		1.0	
Mixed	1.6	1.2–0.8	1.2	0.9–1.7	1.5	0.9–2.5	1.4	0.9–2.3
Black	1.1	0,7–1.7	0.8	0.5–1.2	1.7	0.9–3.2	1.3	0.7–2.5
**Income**								
≤1	1.0		1.0		1.0		1.0	
2<-3	0.6	0.4–0.8	0.6	0.4–0.8	1.1	0.7–1.7	1.1	0.7–1.8
≥3	0.5	0.3–0.7	0.5	0.3–0.8	0.8	0.4–1.7	0.8	0.4–1.7
**Schooling**								
0–3	1.0		1.0		-	-	-	-
4–7	0.6	0.3–1.0	0.6	0.3–1.0	-	-	-	-
8–10	0.7	0.4–1.1	0.8	0.5–1.3	-	-	-	-
>10	0.8	0.5–1.2	1.0	0.6–1.6	-	-	-	-

^a^—Salvador; Ilhéus; Itabuna; Jequié; Fortaleza; Campo Grande.

**Table 3:** Odds ratio crude* and adjusted obtained by logistic regression for the association between Dengue Hemorrhagic Fever and select comorbidities of ≥ 15 years old residents in six municipalities of Brazil 2009–2012.

Chronic Disease	OR Crude (IC a 95%)	OR Adjusted (IC a 95%)
**Hypertension**		
**No**	1.0	1.0
**Yes**	1.3 (0.9–1.7)	1.6 (1.1–2.1)
**Allergy**		
**No**	1.0	1,0
**Ye s**	1.2 (0.8–1.6)	1.1 (0.8–1.6)
**Food Allergy**		
**No**	1.0	1.0
**Yes**	1.3 (0.7–2.5)	1.0 (0.5–2.2)
**Respiratory Allergy**		
**No**	1.0	1,0
**Yes**	1.0 (0.7–1.5)	1.1 (0.7–1.6)
**Skin Allergy**		
**No**	1.0	1.0
**Yes**	1.8 (1.1–3.0)	1.8 (1.1–3.2)
**Diabetes**		
**No**	1.0	1,0
**Yes**	1.0 (0.6–1.8)	1.2 (0.7–2.3)
**Diabetes with Hypertension**		
**No**	1.0	1.0
**Yes**	1.0 (0.5–2.0)	1.2 (0.6–2.5)
**Asthma**		
**No**	1.0	1.0
**Yes**	1.4 (0.7–3.1)	1.1 (0.4–2.6)

*Adjusted for income, skin color and schooling.

There was no statistically significant associations in those younger than 15 years.

## Discussion

Adults with DF and preexisting arterial hypertension or skin allergy were, respectively, 1.6 and 1.8 times more likely to progress to DHF. With respect to hypertension, this risk was marginally stronger in individuals who were not receiving treatment. This association was observed previously in Brazil in black individuals [10], while in Singapore hypertension was a risk factor for developing DHF when associated with diabetes [11].

The mechanism by which arterial hypertension might increase the risk of progression to DHF is not well understood, although the association is plausible. Hypertension leads to endothelial dysfunction and vascular damage, promoting inflammatory activation of the endothelium, changing the regulation of vascular tone and flow [14]. There is some evidence that C-reactive protein (CRP) [15], commonly elevated in hypertension, can promote detrimental effects on the vascular wall, inducing endothelial dysfunction and reducing nitric oxide bioavailability, which also operates in tissue coagulation [16]. This could contribute to increased vascular permeability and coagulopathy, loss of fluid that may progress to hypovolemic shock, characteristic of DHF/DSS [17]. In addition, in animal models treatment of hypertension with Angiotensin-II Receptor Blockers (ARBs) reduces the level of inflammatory activation in vessels, because this drug can reduce the circulating levels of some inflammatory mediators and CRP [16]. This is consistent with our findings that patients receiving treatment for arterial hypertension were less likely to develop DHF than patients who were not. We do not have information about the type of drugs used by the patients, so this association could not be investigated further.

In addition, we do not have in the study information or samples that allow further investigation of the mechanism for the association between FHD and skin allergy. Rather than proposing hypothesis for this association, we recommend that future studies are designed and conducted to answer this specific question.

The study had some limitations: we did not have information on previous history of dengue (so could not control for whether they had a heterologous re-infection); we did not explore immunological mechanisms. Information on co- morbidities were reported rather than abstracted from previous clinical records, and although it is unlikely that this was over diagnosed (as we required evidence of prescription or medication package) it is possible that some allergic patients were classified as non-allergic if they were not able to provide evidence of treatment. Although there was plenty of power to explore the main hypothesis, power might have been limited to investigate risk factors in under 15 years, and separately in each of the 6 cities. In addition, it is also possible that the multi-center nature of the study introduced specific issues that might have limited the investigation of dengue pathogenesis. Finally, cases and controls were from selected in public health units that attended dengue cases in the course of epidemics, so although this would not have led to selection bias (as cases and controls came from the same population) our findings may not be generalizable to wealthier populations that would have been seen in private hospitals.

Although gaps remain in our understanding of determinants of DHF, we believe that there is now sufficient evidence of the increased risk of progression to DHF for us to recommend the physicians attending patients with dengue fever who have a history of hypertension or skin allergies should keep the patients for observation to be able to intervene in a timely fashion and avoid death. We recommend that research be conducted to understand the pathogenesis DF/DHF/DSS, focusing on the influence of the immune system, especially the role of cytokines, such as IL-10 on the evolution from DF to DHF. In particular, we recommend continuing to investigate the influence of the immune system in order to possible the identification of immunological biomarkers that can function as prognostic indicators of progression.

## Supporting Information

S1 Checklist STROBE checklist. Found at: doi:10.1371/journal.pntd.0000699.s001 (0.20 MB RTF) http://www.strobe-statement.org/fileadmin/Strobe/uploads/checklists/STROBE_checklist_v4_combined_PlosMedicine.pdf
(DOC)
